# Molecular Dynamics in Two-Dimensional Supramolecular Systems Observed by STM

**DOI:** 10.3390/ma3084252

**Published:** 2010-08-06

**Authors:** Shinobu Uemura, Ryota Tanoue, Neval Yilmaz, Akihiro Ohira, Masashi Kunitake

**Affiliations:** 1 Graduate School of Science and Technology, Kumamoto University, 2-39-1, Kurokami, Kumamoto 860-8555, Japan; E-Mails: shinoue@kumamoto-u.ac.jp; 095d8336@st.kumamoto-u.ac.jp; 2 JST-CREST, 744 Moto-oka, Nishi-ku, Fukuoka 819-0395, Japan; E-Mail: nevaly@kumamoto-u.ac.jp; 3 Research Institute for Ubiquitous Energy Devices, National Institute of Advanced Industrial Science and Technology, 1-8-31, Midorigaoka, Ikeda, Osaka 563-8577, Japan; E-Mail: a-oohira@aist.go.jp

**Keywords:** scanning tunneling microscopy (STM), dynamics, adsorption-induced, self-crystallization, self-assembly

## Abstract

Since the invention of scanning tunneling microscopy (STM), 2D supramolecular architectures have been observed under various experimental conditions. The construction of these architectures arises from the balance between interactions at the medium-solid interface. This review summarizes molecular motion observed in 2D-supramolecular structures on surfaces using nanospace resolution STM. The observation of molecular motion on surfaces provides a visual understanding of intermolecular interactions, which are the major driving force behind supramolecular arrangement.

## 1. Introduction

Scanning tunneling microscopy (STM) was invented in 1982, and has allowed significant advances in various scientific fields through facilitating the real space imaging of molecules on surfaces [1]. Molecular imaging elucidating the arrangements in arrays, orientations and even intramolecular structures has been achieved in air [2], ultrahigh vacuum (UHV) [3] and solution [4,5,6,7,8]. Construction and in-space visualization of supramolecular adlayers (adsorbed layers) on well-defined single crystal surfaces have received significant attention in the last decade. Coupling supramolecular chemistry and scanning probe microscopy (SPM) allows the visual consideration of molecular self-assembly in sub-molecular space.

The adlayer structures of organic molecules are generally controlled by the balance between adsorbate-substrate (epitaxial) interactions and intermolecular ones. Molecular adsorption on well-defined single crystal surfaces has often been considered from a static crystallographic point of view, *i.e.*, simply as a matter of surface chemistry. In such systems (known as epitaxial adsorption or epitaxy) predominantly static structures have been discussed with reference to properties like adlayer commensuracy and adsorbate orientation. Conversely, the self-assembly of sophisticated and highly ordered supramolecular adlayers has been predominantly discussed in terms of intermolecular interactions rather than epitaxial arrangements in surface chemistry [9,10]. In the case of weak or mild adsorption (typically physisorption), molecules on surfaces are relatively unrestrained by the substrate lattice, thus isolated molecules on surface can move spontaneously to reach a thermodynamically stable adlayer structure. In other words, artistic 2D-supramolecular systems are regulated by intermolecular interactions and subsequent molecular motion in weak adsorption systems.

In this review, we focus on molecular motion in 2D-supramolecular structures on surfaces visualized by STM with nanoscale resolution. Observed dynamic motion of molecules on surfaces provides a “visual” understanding of intermolecular interactions, which are the major driving forces behind supramolecular arrangement.

## 2. Thermodynamic Equilibrium for Molecules on a Substrate

The basic concept of molecular dynamics on surfaces, and self-assembly induced by intermolecular interactions, will first be explained using a classical theory according to colloidal chemistry in solution rather than surface science. STM imaging depends on the interface in question, which generally falls into one of the following categories: (1) solid–UHV, (2) solid–liquid in an apolar solvent, and (3) solid–liquid in electrolyte solutions. In UHV systems, molecular adlayers are prepared by sublimation, and the molecular dynamics on substrate are frequently described as a function of temperature. For *in-situ* STM observation the adlayers are generally prepared by spontaneous adsorption from solution phases, and STM imaging is conducted at the solid–solution interface in presence of sample molecules. Here, we largely focus on molecular adlayers prepared by physisorption and then observed at an aqueous or organic solvent–solid interface.

**Figure 1:**
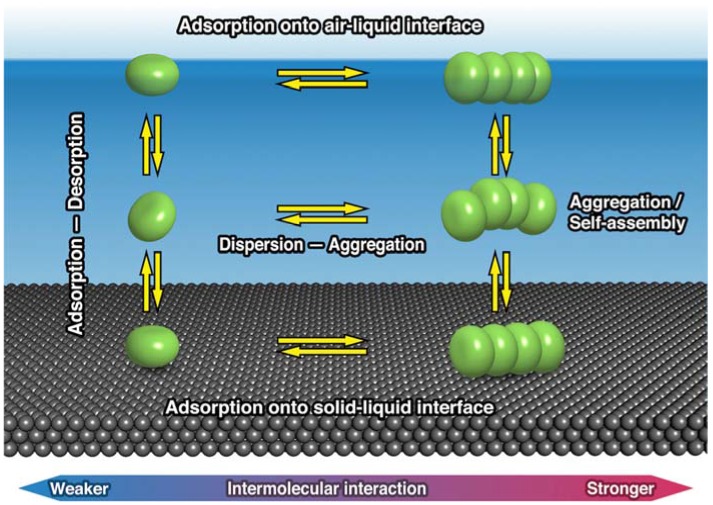
Thermodynamic equilibriums between a homogeneous solution, an air–liquid interface and a solid–liquid interface.

Among self-assembled monolayers (SAM), the system based on thermodynamically reversible adsorption (physisorption) should be differentiated from the chemisorption systems (e.g., thiols on Au). To understand the molecular behavior on the surface including individual molecular motion and self-assembly, the phase equilibrium at the solid–liquid interfaces must be considered. Figure 1 shows a schematic phase equilibrium diagram for molecules in the solution phase and at solid–liquid and liquid–air interfaces. Phase equilibrium in solution can be roughly classified into adsorption–desorption and dispersion–aggregation processes, which in Figure 1 are expressed as vertical and horizontal arrows, respectively. Reversible adsorption controls molecular distribution at an interface based on the adsorption–desorption equilibrium. The phase transition between monomeric dispersion and aggregation (based on the equilibrium) also exists at the solid–liquid interfaces. This is similar to molecular aggregation within the solution phase, examples of which include micelle, bilayer and liquid crystal formation. The equilibrium at the solid–liquid interface is primarily governed by temperature and surface concentration. Solution conditions, such as solvent polarity and sample concentration, also influence the equilibrium, because these change the solubility of the adsorbate. The affinity of the adsorbate the substrate strongly influences the surface concentration and therefore the equilibrium distribution. The contribution of adsorption strength (adsorbate–substrate interaction) will be discussed later.

In a surface aggregation state, highly ordered molecular adlayers with supramolecular arrangements are frequently formed as a result of 2D-crystallization or self-assembly on surfaces. Molecules generally construct a thermodynamically stable structure, normally a closely packed adlayer with the highest surface coverage.

**Figure 2:**
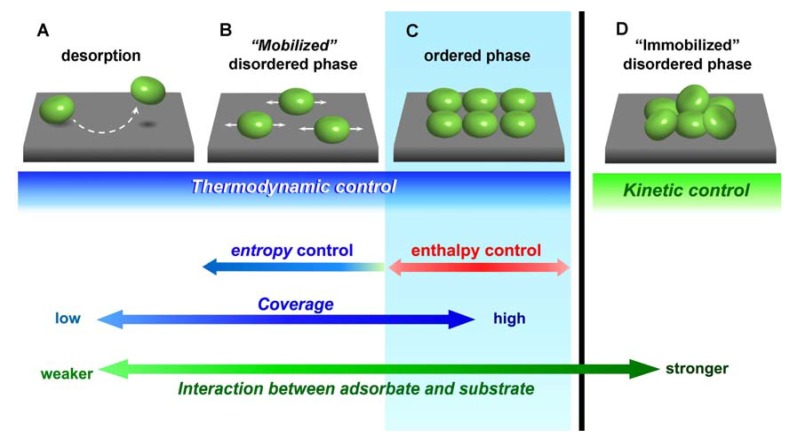
Four isothermal adsorption states against adsorption strength.

Control of adsorption strength is one of the most crucial points for constructing highly-ordered adlayers. Isothermal adsorption at the solid–liquid interface can be classified into four states related to adsorption strength (adsorbate–substrate interaction), as shown in Figure 2. The states from A to C, in which the adsorbate–substrate interaction is relatively weak or has only van der Waals interactions, have adsorption behavior governed by the thermodynamic equilibrium between adsorption and desorption. As mentioned above, the solution conditions also strongly contribute to the adsorption–desorption equilibrium. State A corresponds to a situation where no molecules are adsorbed because of the very weak adsorbate–substrate interaction and the high adsorbate solubility. Thus, equilibrium tends toward the solution phase.

In state B molecules adsorb on surfaces macroscopically due to the stronger interaction with the substrate. Molecules are not statically immobilized onto substrates due to a rapid adsorption–desorption equilibrium, and may be translocated on the substrate by weak intermolecular interactions. In this state, it is difficult to observe such rapid motions of dynamically isolated molecules using SPM because of the relatively slow scanning speed, although molecules on the substrate can be monitored by spectroscopic techniques as an average of the surface concentration.

In state D, the adsorbate–substrate interaction is very strong, and chemisorption generally occurs with the formation of covalent or coordination bonds. This restriction of molecular motion leads to the formation of an epitaxial adlayer with low commensuracy. Highly-ordered adlayers of small aromatic molecules are known to form in an epitaxial fashion on reactive metal surfaces such as Pt and Rh. Such molecular adsorption has been visualized by STM in UHV and in aqueous solution under electrochemical control. Very strong adsorbate–substrate interactions restrict molecular motion on the substrate, the adsorption–desorption equilibrium is then extremely slow, and the adsorption is irreversible. Kinetically controlled adsorption is crucial for adlayer structures, and will be discussed later. Disordered adlayers or randomly adsorbed molecules are frequently observed, as opposed to ordered structures, and this could be due to limited molecular diffusion on the surface. Small chemisorbed molecules can move by hopping between neighboring adsorption sites.

State C is where highly ordered molecular adlayers are constructed, and this is based on intermolecular interactions rather than interaction with the substrate. In these thermodynamically controlled ‘mild’ adsorption conditions, the adsorption–desorption equilibrium and lateral diffusion of molecules on the surface are highly maintained. Self-organization at solid–liquid interfaces is then achieved by intermolecular interactions as the driving force of 2D-ordering (2D-crystallization). The resemblance between self-organization in homogeneous solutions and self-assembly (adsorption-induced self-crystallization or self-organization) on surfaces is worth pointing out (Figure 1); both correspond to a thermodynamic meso-phase achieved by establishing a suitable interaction balance.

Transitions between states B and C have been frequently observed as order-disorder transitions [11,12]. These transitions reveal the boundary between entropic and enthalpic controls at a given surface concentration, and correspond to the critical micelle concentration for dispersed aggregation. At surface coverage less than the critical micelle concentration, the intermolecular interaction is repulsive. When the surface concentration increases beyond this concentration, attractive intermolecular interactions become the driving force for the self-organization on the surface. In fact, the hydrophobic effect, which is a major driving force of micelle formation, is believed to be an entropic effect. Even so, micelle formation also complies with Gibbs equation, dG = dH – TdS, in terms of temperature dependency. The formation and deformation of micelles is controlled by the direction of Gibbs free energy, negative (entropic) to positive (enthalpic), respectively.

Chemisorption and physisorption are the extreme adsorption conditions for adlayers on well-defined surfaces. The former is generally an irreversible epitaxial adsorption with the resultant structure strongly regulated by the underlying substrate lattice. The atomic lattice structure and kinetic adsorption process strongly influence the adlayer structure. The latter condition is less intense and is based on a dynamic equilibrium, and the adlayer structure formed is strongly influenced by the intermolecular interactions. The adlayer structure is dynamic and can change according to the solution condition. There is no well-defined border between chemisorption and physisorption, or between epitaxy and adsorption-induced self-crystallization. In a realistic adsorption system, the adsorbate-substrate interaction should enforce adsorption even in a thermodynamically reversible adsorption system (except for a van der Waals interaction or covalent bond). The balance between epitaxial restriction and intermolecular interactions will dictate whether epitaxy or dynamic self-assembly on surface will occur dominantly or simultaneously.

Adsorption at state D is kinetically rather than thermodynamically regulated. Thus, kinetic factors are also crucial in controlling the adlayer structure formed, including epitaxially ordered adlayers. Thermal treatment from high to low temperatures (annealing) is commonly applied to 2D- and 3D-crystal structures to diminish defects and improve overall crystal quality. From the viewpoint of surface molecular dynamics, this corresponds to a change from adsorption state B or C to state D. Electrochemical potential management is also frequently used as a wet process to form epitaxial adlayers. This would correspond to a change from an adsorption state of A or B to state D. Details of this will be discussed later.

Molecular adlayers based on weak adsorbate–substrate interactions are offen reported as “van der Waals epitaxy” [13] in vapor deposition studies. When the adsorbate–substrate interaction is very weak (typically consisting of only van der Waals force interaction), highly ordered molecular layers can be formed by sublimation, despite a large lattice mismatch between the molecular adlayer and the substrate. Ward and co-workers have discussed in detail epitaxial molecular organization on solid substrates based on various UHV-STM studies [14]. They also refer to the significance of the balance between overlayer–substrate and molecule–molecule energies.

## 3. Relation between Molecular Motion and Adsorption Strength

**Figure 3:**
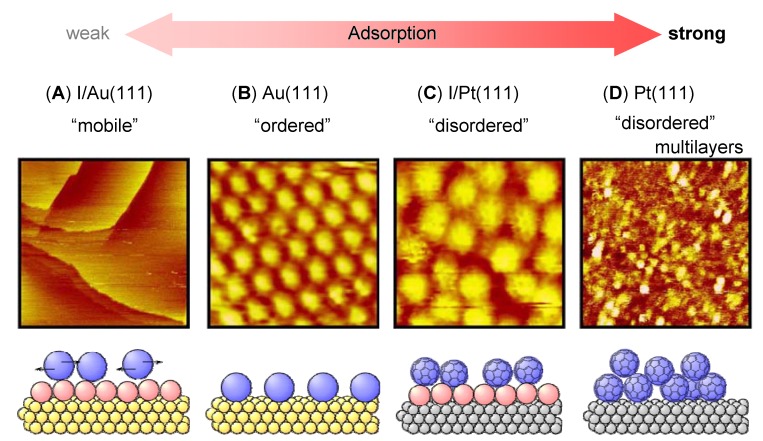
Molecular motion of fullerene C_60_ on various substrates. Adapted with permission from reference [18]. Copyright 2004 American Chemical Society.

It is well-known that the substrate has a direct effect on the 2D arrangements of adsorbed molecules and molecular motions. The effect of various single crystal metal substrates on organic molecular adlayers has been previously discussed in detail [15,16]. The effect of substrate on fullerene adlayer morphology observed by STM is a good example to examine this relationship (Figure 3). C_60_ molecules on Au(111) surfaces form 2D self-assembled structures with high uniformity, which may rotate at room temperature. When an iodine modified Au(111) [I/Au(111)] surface is used as the substrate, it is difficult to observe the self-assembled structure of C_60_ molecules by STM [17,18,19]. The reason for the visualization of only the substrate surface in aqueous solution in Figure 3A is the sweeping of C_60_ molecules by the STM tip due to the weak interaction between C_60_ and I/Au(111) surface. In contrast to C_60_ on I/Au(111), C_60_ molecules on the bare Au(111) surface formed 2D self-assembled structures with high uniformity, after the electrochemical removal of iodine layers (Figure 3B). This structure was similar to the adlayers prepared by the vapor deposition and the direct transfer of Langmuir films on a bare Au(111) surface, but had fewer defects at the phase boundaries. C_60_ molecules adsorbed randomly on Pt(111) had their features veiled due to the excess adsorption (multilayers) on the substrate (Figure 3D).

C_60_ molecules also adsorbed randomly on an iodine modified Pt(111) (I/Pt(111)) surface, but were observed more clearly compared with the features on Pt(111) because of less adsorption (Figure 3C). Darker hollows in the bright spherical features of C_60_, similar to those observed at low temperature (Figure 9), were easily observed in the image on I/Pt(111) surface. It was difficult to observe any features in the C_60_ spots on Pt(111) surface. This visualization of the C_60_ internal structure indicated that the molecular motion of C_60_ on I/Pt(111) could be stopped by the strong interaction between the molecule and substrate at room temperature in solution. In addition to I/Pt(111) and Pt(111) surfaces, this rotation could also be stopped at room temperature in UHV on other substrates including Pb-covered Si(111) surface [20]. Using uniform 2D structures of C_60_ on Au(111), the motion of other planar molecules could be controlled by manipulating interactions and temperature [21,22].

## 4. Dynamics of 2D Supramolecular Structures

As mentioned above, 2D supramolecular structures can be obtained through suitable interactions between molecule, substrate, and medium. Molecules in the 2D supramolecular structure are ordered on the substrate, but not immobilized, and the lateral molecular motion (diffusion) is decreased by the surrounding envionment. *In-situ* STM observations of dynamics of 2D structures, such as construction and phase transition, have been frequently reported.

### 4.1. Self-ordering Processes Induced by Adsorption from Solution [12,23]

**Figure 4:**
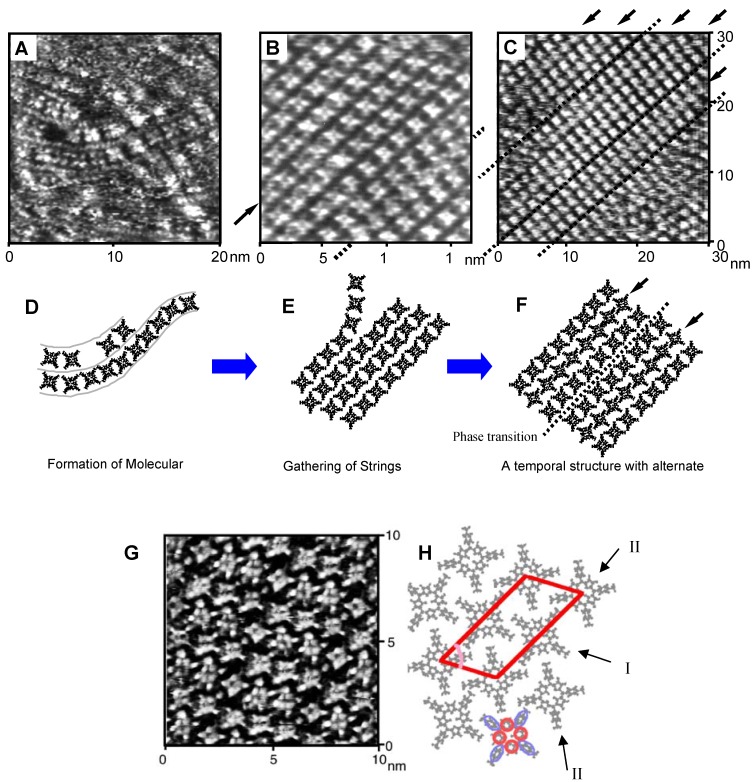
*In situ* STM images and corresponding models of the self-ordering process of TMPyP on I/Au(111) captured at each step of adsorption and ordering. Adapted with permission from reference [12]. Copyright 1995 American Chemical Society.

A typical example of dynamic processes upon self-assembly on the surface is the *in situ* STM observation of adsorption and self-ordering processes of the porphyrin derivative (TMPyP) on an I/Au(111) surface in aqueous solution. To observe self-ordering process, a solution was injected into the STM sample cell during *in-situ* observation. The iodine adlayer became progressively less clear with time, and adsorbed TMPyP molecules became visible following the formation of temporal structures (Figure 4). Prior to the surface becoming completely covered with a highly-ordered closed packed adlayer, several interesting temporal structures were formed as steps in the self-ordering process.

Figure 4A shows an STM image collected at a very early stage of adsorption of TMPyP. Many flat-lying TMPyP molecules, which can be recognized as squares with four bright spots at the corners, were aligned one-dimensionally, forming molecular chains. Adjacent molecules in each chain were aligned with a side-by-side configuration. Formation of molecular chains would involve weak coordination of shared counter anions. During continuous imaging (after image acquisition), the relative location and shape of the flexible chains changed rapidly. Slow moving molecules restricted by ordering were observed, but not isolated.

During the ordering process, twisted 1D molecular chains gathered and then assembled into 2D ordered arrays in a relatively small domain as a transitional phase (Figure 4B and E). Arrays possessed a similar side-by-side configuration along a molecular row indicated by an arrow, as a memory of the strings from the previous step. It was surprising to find that in a very short period of time all molecules along rows marked by arrows were shifted by half a position (Figure 4B and C), and were rotated by nearly 45° with respect to the unchanged molecular rows (Figure 4G and H). Figure 4C was captured in the phase transition, 12 seconds after Figure 4B at the same location.

A spontaneous phase transition occurred within the image capture interval time. Molecular rotation in the rows should occur cooperatively. The final array structure, which was thermodynamically the most stable structure, appeared soon after acquisition of Figure 4C. Further rearrangement took place in a short period of time to form the final stable structure as shown in Figure 4G. Increasing the surface coverage may have been the driving force for the phase transition.

Once the final structure appeared in isolated domains, these domains expanded with time and molecules within the disordered phase were progressively incorporated into the ordered domain, as shown in Figure 4C and F. Arrows in Figure 4C indicate a single molecular defect located at the cross point of two phase boundaries, caused by the rotation. 1D-strings were also frequently observed connected with the edges of ordered domains. This confirmed that the phase transition from 1D-strings to 2D-arrays with the final alternate arrangement and expansion of 2D-arrays occurred simultaneously. This demonstrated that the surface diffusion of adsorbed molecules is a key factor for the ordering process.

### 4.2. Ostwald Ripening

The size of ordered domains is generally kinetically controlled, despite the overall adlayer structure being thermodynamically controlled by intermolecular interactions. To form high quality adlayers with low defect numbers and large ordered domain sizes, a slow supply of molecules to the surfaces would be advantageous, similar to the case of bulk crystallization controlled by nucleation and crystal growth. Rapid contact from a high concentration sample solution and corresponding rapid nucleation would lead to the formation of mosaic structures with small ordered domains. Eliminating domain boundaries minimizes the free energy and leads to the growth of larger islands at the expense of smaller ones (2D Ostwald ripening) [24,25,26,27,28].

**Figure 5:**
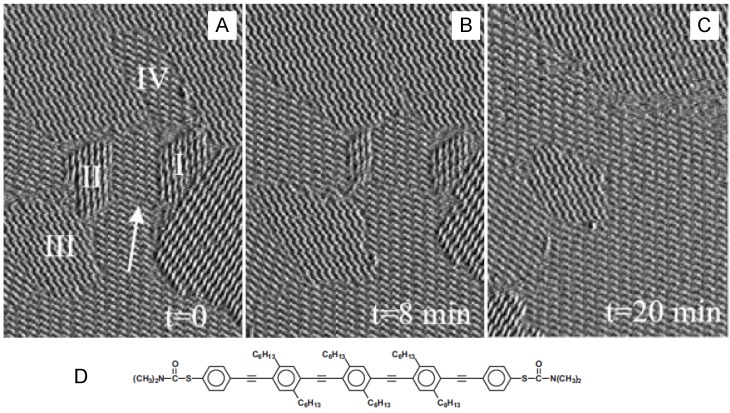
Sequential STM images (A-C) of a phenylene ethynylene derivative (D) at the solution–HOPG interface. Reproduced with permission from reference [25]. Copyright Wiley-VCH Verlag GmbH & Co. KGaA.

The first STM observation of Ostwald ripening of 2D organic molecular structures was reported by Rabe and co-workers [24]. Rod-like molecules with long alkyl chains such as oligothiophene and oligophenylene ethynylene formed 2D self-assembled structures with many small ordered domains at the solution–HOPG (highly oriented pyrolytic graphite) interface [25], as shown in Figure 5.

These ordered domains consisted of identical molecules in different orientations, and the domain numbers and features changed over time from the domain edges. The smaller domains decreased in size and eventually disappeared. Compared with smaller molecules such as anthraquinone, the rate of Ostwald ripening for oligothiophene derivatives is very slow and the process can be followed by STM [24]. The primary factor for the coarsening phenomena is the interplay of intermolecular and interfacial interactions, and the solution condition and temperature are also important factors affecting the rate of Ostwald ripening. The result indicated that molecular motion at the solution–solid interface was governed by the balance of interactions between solid and solution phase molecules. The dynamics of ordered structures on a nanometer scale (not strictly Ostwald ripening) have also been observed by STM at the solution–HOPG interface [27].

### 4.3. Two-Dimensional Phase Transition

#### 4.3.1. Single-molecular species

Prior to the invention of STM, order-disorder phase transitions in organic molecular layers at interfaces were investigated by *in-situ* spectroscopic and *ex-situ* UHV techniques. The first *in-situ* STM observation of an order-disorder phase transition for a 2D self-assembled structure was reported by Rabe and Askadskaya [29]. They made *in-situ* STM observations of long alkane chains (24-192 carbon atoms) on HOPG surfaces in solutions of phenyloctane or molten long alkane. The appearance and disappearance of lamellar structures indicated a 2D-phase transition at higher and lower temperatures, respectively, relative to the critical phase transition temperature (2D-melting point). Similar to solid–liquid phase transitions, interfacial order-disorder phase transitions correspond to the boundary between enthalpic control and entropic control. Thus the transition was regulated by temperature and density (concentration).

For thermodynamically constructed adlayers, the order-disorder transition can be induced by altering surface concentration (coverage). This represents a phase transition between state B and C in Figure 2. Surface concentration is regulated by adsorption strength and solution conditions such as concentration and pH. Adsorption strength determines the adsorbate distribution between the sample and substrate, as demonstrated in Figure 3.

For an electrochemical-dependent system in an electrolyte solvent, the adsorption strength and therefore surface concentration can be changed by applying a potential to the substrate. Surface charge density at the electrochemical interface can be controlled continuously and preciously. This is one of the biggest advantages of electrochemical STM systems in solution compared to an *in-situ* STM system in a non-electrolyte solvent. Phase transition induced by an applied potential was first reported by Tao and Cunha [11]. 2,2’-Bipyridine (22BPY) formed parallel stripes with a high packing density on Au(111) at 0.4 V *vs.* SCE. 22BPY was in a standing orientation with nitrogen atoms in contact with Au(111) surface, and the stacked neighboring 22BPY molecules were shifted slightly in the lateral direction. When the substrate potential (working electrode) was altered to lower the surface charge density, 22BPY molecules became stacked as randomly oriented polymeric chains. This was attributed to the surface charge-induced increase in the concentration of the adsorbed molecules. Following this report, phase transitions for adsorbed cyclodextrins [30,31,32], trimesic acid (TMA) [33], porphyrins [34], and other mixed molecules [35,36,37] were reported.

**Figure 6:**
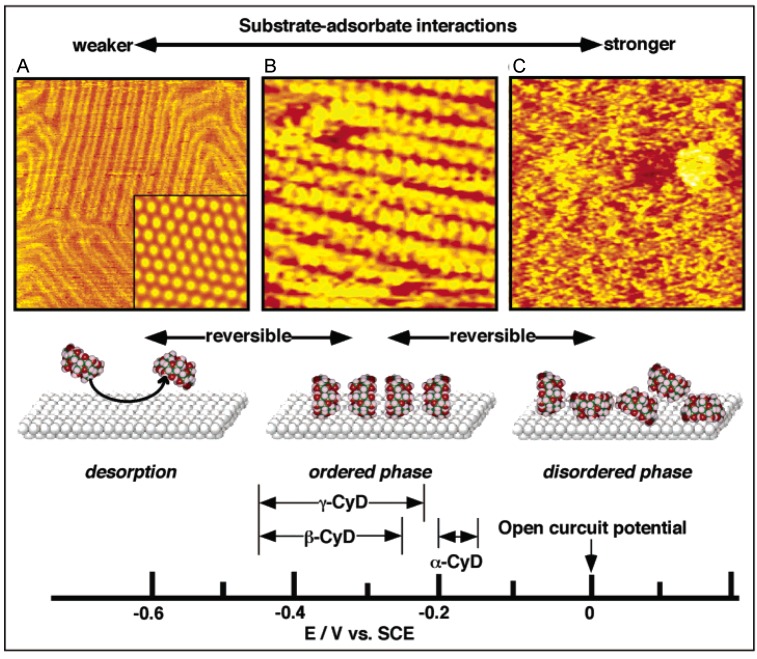
*In-situ* STM images and a schematic illustration of CyDs at the aqueous solution–Au(111) interface controlled by electrochemical potential. Reprinted with permission from reference [32]. Copyright 2003 American Chemical Society.

Electrochemical potential management is a sophisticated technique allowing the interaction balance to be moderated to produce 2D-supramolecular structures. A typical example is shown in Figure 6 [30,31,32]. In host-guest and supramolecular chemistry, cyclodextrins (CyDs) are water-soluble host molecules possessing the ability to capture hydrophobic guests in their interior hydrophobic cavity.

At approximately open circuit potential (OCP), CyDs adsorbed randomly onto Au(111) with a high density (disordered phase, Figure 6C). As the potential became more negative, the density of adsorbed CyDs decreased, and finally no CyDs could be observed on the surface by STM (Figure 6A). Many neutral molecules undergo desorption from various negatively polarized substrates. It is generally accepted that the adsorption of neutral molecules on polarized surfaces is unfavorable. Counter-ions and water molecules from the electrolyte solution approach polarized surfaces to compensate for the surface charges, and the adsorption of counter-ions and water prevents that of neutral molecules. The adsorbate surface coverage was controllable as a function of electrode potential.

At certain mid-range potentials, it was found that ordered molecular arrays with nanotube-like structures, which resembled polyrotaxanes self-constructed by threaded polymers in solutions, formed spontaneously on the substrate (Figure 6B). The dominant driving forces for the tube formation was intermolecular hydrogen bonding between hydroxyl groups on the same side faces of CyD. Identical intermolecular interactions are involved in the formation of polyrotaxanes.

The three adsorption phases: disordered adlayer, ordered adlayer with nanotubes, and desorption, were reversibly controlled by the electrode potential. The phases obviously correspond to the isothermal adsorption states in order of adsorption strength, as shown in Figure 2. The observation of various dynamic phase transition processes induced by changing potential (e.g., the formation and destruction of nanotubes) has been achieved by STM. Nanotube structures were formed from all types of CyDs, namely α-, β-, and γ-CyDs, at different potential regions as indicated by arrows on the potential scale in Figure 6. The difference of preferable potential for nanotube-like structure of each CyD was established on the thermodynamically delicate balance between intermolecular interaction and epitaxial interaction.

**Figure 7:**
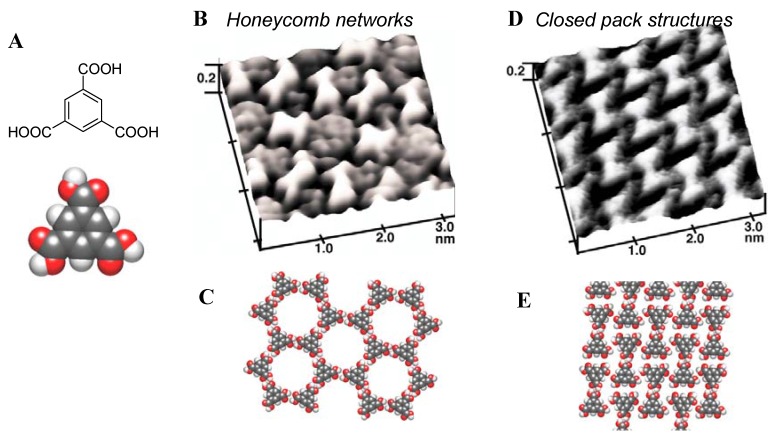
Chemical structure (A), STM images (B and D) and schematic illustrations (C and E) of TMA order-order transitions at the aqueous solution–Au(111) interface under the electrochemical control. Reproduced from reference [33] by permission of The Royal Society of Chemistry.

In addition to order-disorder transitions, order-order transitions were revealed under delicate surface potential control [33]. A closed packed structure with the highest surface coverage is thermodynamically advantageous in many adlayer systems. However, a few adsorption systems showed several different ordered structures and order to order phase transitions. Such systems are very interesting as they represent a transformable self-organized supramolecular structure. An example is TMA adsorbed on Au(111). Adsorbed TMA generally revealed three phases: desorption, ordered adlayer, and disordered adlayer. Ordered adlayers were observed in the mid-potential range between the desorption and disordered adlayer phases, similar to the potential-dependent adsorption behavior of CyDs on Au(111). It was found that two ordered phases, a honeycomb network and a closed pack array, appeared in the mid-potential region, as shown in Figure 7.

Both phases were reversibly controlled by modifying potential. The honeycomb network was observed at more negative potentials than the closed pack phase. In the honeycomb network, TMA molecules were connected to each other via hydrogen bonding between carboxylic acid moieties. Increasing the adsorbate–substrate interaction induced transformation from the honeycomb network to the higher coverage closed pack structure by cutting off intermolecular hydrogen bonds in the network. This suggests that strong specific intermolecular interactions, which can overcome the thermodynamic energy loss from low surface coverage, aided the construction of the non-closed packed network structure with a cavity. This behavior was also observed by UHV-STM [38]. In addition to the order–order transition of achiral rod-like molecules, other 2D chiral structures have also been constructed [39].

**Figure 8:**
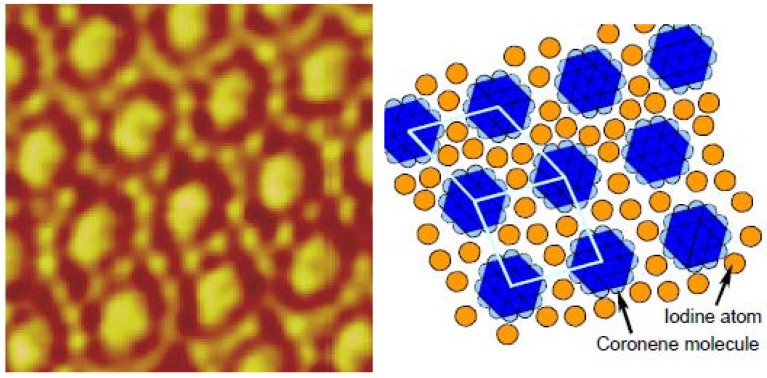
STM image and tentative model of coronene and iodine co-adsorption at the aqueous solution–Au(111) interface under the electrochemical potential control. Reproduced from reference [40] by permission ECS―The Electrochemical Society.

Successive structural change of ordered adlyers dependent on electrochemical potential control was also observed for coronene–iodine co-adsorption on Au(111) [40]. A pseudo-hexagonal iodine lattice was observed at the OCP. The cathodic desorption of iodine and replacement with coronene adlayers occurred at potential less than 0.10 V *vs.* RHE. In the mid-potential range 0.20–0.45 V *vs.* RHE, co-adsorbed adlayers were observed. Figure 8 shows a typical *in-situ* STM image and the corresponding model of typical co-adsorbed coronene–iodine adlayers. The large and small spots correspond to a coronene molecule and an iodine atom, respectively. The three phases (coronene, co-adsorption and iodine adlayers) were reversibly obtained, indicating that the co-adsorbed adlayer was a thermodynamically-controlled phase, not an unstable temporal state.

Although the observed array was very stable at each potential, the adlayer structures changed with the potential. The various observed structures of co-adsorbed adlayers were essentially based on the combination of a quasi-square unit cell and an oblique unit cell, as marked in Figure 8. The results showed that negative electrochemical potential led to a high proportion of oblique unit cells in the adlayer, suggesting higher coronene and lower iodine coverage.

Thus, various unique supramolecular structures can be managed with surface potential control. The surface charge potential is based on the adsorption-induced self-crystallization principle, and is an effective factor for constructing multicomponent supramolecular structures. Furthermore, 2D phase transition by electrochemical potential allows a switching of the structure by external stimuli, and could be applied as a sensor or switch.

#### 4.3.2. Competitive physisorption dynamics

Dynamic processes with a mixture of multiple molecules have also been reported. As mentioned above, the potential range for nanotube formation differed for each CyD. When α- and γ-CyDs were in the same solution, the replacement and formation of each CyD nanotube could be observed because their respective potential ranges did not overlap [32].

**Figure 9:**
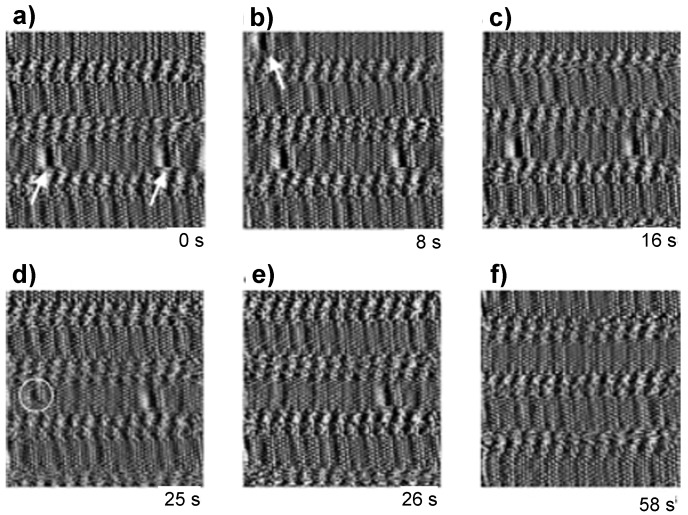
Dynamic processes during co-adsorption of perfluorinated and non-fluorinated isophthalic acid derivatives on HOPG. Reproduced with permission from reference [41]. Copyright Wiley-VCH Verlag GmbH & Co. KGaA.

Competitive physisorption dynamics has also been reported at the organic solution–substrate interface. De Feyter and De Schryver reported the dynamics of co-adsorbing isophthalic acid derivatives and solvents on HOPG [41]. In a mixture of semi- and non-fluorinated isophthalic acid derivatives, dynamic processes of single molecules could be observed by STM due to the differing STM contrast of these derivatives. Fluorinated alkane parts indicated by arrows in Figure 9 were seen as darker bands with respect to non-fluorinated ones, and the single semi-fluorinated molecules could be recognized easily. The fluorinated parts disappeared and were replaced with non-fluorinated molecules with time (Figure 9b-f). This was due to the lower adsorption energy of the fluorinated parts to graphite compared with the non-fluorinated ones, and the small difference in intermolecular interaction energy. It indicated that the co-adsorbed structures might be on a quasi-stable state.

In addition to the above, the replacement of bithiophene derivatives, which was induced by the addition of another derivative with a different alkyl chain length, was reported [42]. This was also due to the higher affinity of the subsequently added thiophene derivative. In summary, competitive physisorption dynamics may be induced by the balance between interactions.

### 4.4. Conformational Changes

**Figure 10:**
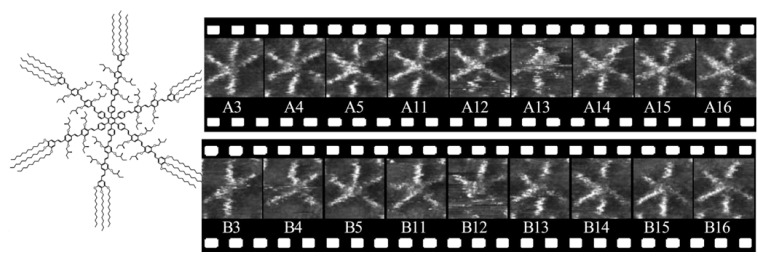
Dynamic processes of a hexapod molecule at the solution–HOPG interface. Reprinted with permission from reference [43]. Copyright 2009 American Chemical Society.

In developing molecular machines, control of molecular conformations on surfaces is very important. An excellent example of such conformational dynamics was reported by De Feyter and coworkers [43]. A molecular hexapod, which has a benzene core and six oligo(*p*-phenylene vinylene) legs (Figure 10 left), formed 2D crystalline structures at the 1-phenyloctane–HOPG interface predominantly. The molecular features of the adsorbed hexapod were not uniform in the STM images. Molecular appearances fully adsorbed by six legs, and partly adsorbed by four or five legs, were all frequently observed. The lack of adsorption by all legs in some cases was due to rapid adsorption-desorption processes. Figure 10 shows that the number and orientation of legs changed frequently, and such changes could be observed by STM with a time difference between two consecutive frames of about 14 seconds. During self-assembly, small molecules typically undergo similar molecular conformational changes, but the rapid time-scales involved mean that such behavior may not be observed by STM. In contrast, the hexapod is a much larger molecular shape and the phenomena were easily observed with STM. Translational dynamics at the interface was also apparent. These dynamic processes occurred without external stimuli such as light or temperature, which could be due to solvation of oligo(*p*-phenylene vinylene) and alkyl chains; the rapid adsorption-desorption equilibrium of the legs.

Molecular conformational changes induced by external stimuli have also attracted attentions. Changes in 2D self-assembled structures of switching molecules induced by such guest molecules [44,45], pH [46], metal ions [47,48] and light [49,50,51,52,53] have already been observed.

**Figure 11:**
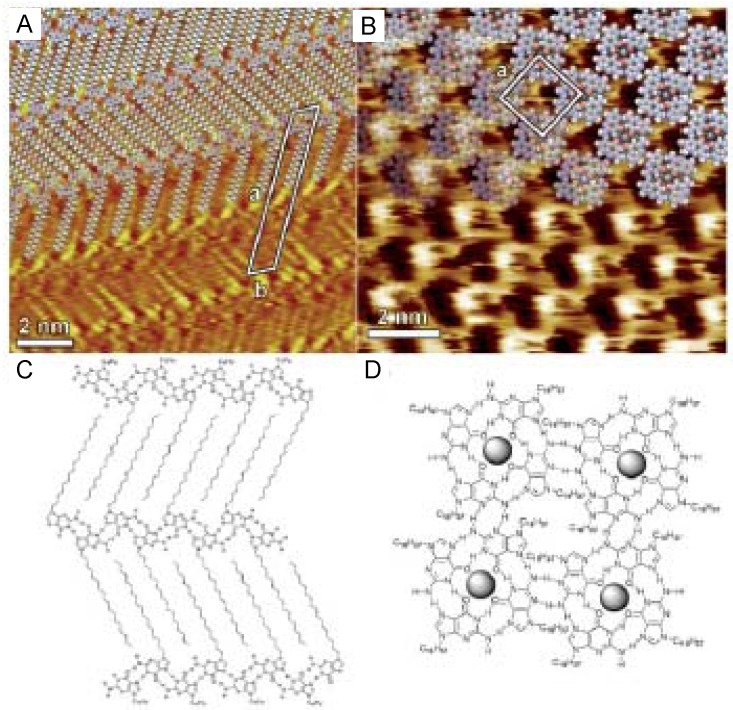
STM images (A and B) and tentative models (C and D) of self-assembled structures of guanines. Without (A and C) and with (B and D) potassium. Reproduced with permission from reference [45]. Copyright Wiley-VCH Verlag GmbH & Co. KGaA.

It is remarkable that reversible 2D structure switching was achieved by adding guest molecules to the solution–solid interface. Samorì and co-workers reported that 2D self-assembled structures of guanine derivative at the solution–HOPG interface could be reversibly switched by the addition of potassium or cryptand (a potassium ion sequentering agent) [45]. Guanine is a nucleobase and forms a quadruplex (G_4_ complex) when induced by metal ions. The guanine derivative had a long alkyl chain and formed 2D head-to-head lamellar structures with multiple hydrogen bonds at the free potassium solution–HOPG interface (Figure 11A). When potassium picrate solution was added to the solution–HOPG interface, the lamellar structures disappeared and cyclic structures induced by the potassium ion were formed with G_4_-based architecture (Figure 11B). Potassium ions could be removed from the G_4_ complex by the addition of cryptand, and the lamellar structures were then reconstructed at the interface. When trifluoromethanesulfonic acid was added to thus releasing the potassium ions from the cryptands, cyclic G_4_ complexes were again observed at the interface. This reaction could be occurred in a bulk solution rather than at the solid–liquid interface.

The ability of guest molecule addition to allow structural switching is due to the reversibility of 2D structures at the interface. The equilibrium distribution was controlled by each molecular concentration and is illustrated in Figure 1.

## 5. Molecular Rotations in Arrays

Molecular building blocks in 2D supramolecular structures constructed via surface diffusion are confined but not chemically immobilized on the substrate. Molecular perturbation in arrays has observed been frequently at room temperature. A molecule trapped in a cavity in an array can exhibit molecular rotation on the surface. As previously mentioned, temperature is a key factor for controlling the molecular motion, and many research groups have reported STM studies of temperature effects. For example, an isolated thioether anchored onto Au(111) rotated and was observed as a round feature at temperatures of 20–78 K [54,55,56]. At 7 K the molecular vibration was almost frozen and this feature appeared enlongated.

**Figure 12:**
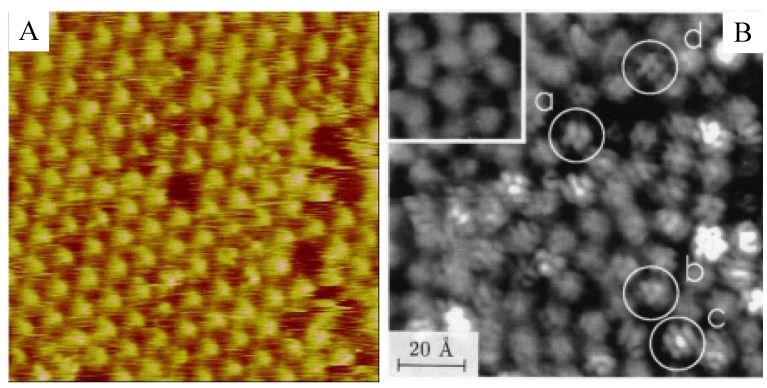
STM images of fullerene C_60_ adlayers on Au(111). (A) The adlayers were prepared by the transfer of L films. STM observation was in aqueous electrolyte solution at room temperature. (B) The adlayers were prepared by the sublimation. STM was conducted in UHV at 4.5 K. The observation at room temperature is shown as the inset. Reprinted with permission from reference [58]. Copyright Springer.

In 2D self-assembled structures, molecular motion such as rotation does not stop, although a molecule’s lateral motion may stop due to hindrance by surrounding molecules. As mentioned earlier, fullerene C_60_ is one of the most commonly studied adsorbed molecules [15]. Regardless of preparation method (e.g., vapor deposition, adsorption from organic solution), C_60_ on Au(111) forms self-assembled hexagonal packed structures, and their structures have been observed by STM in UHV and in solution [17,18,57,58,59,60,61]. Figure 12A shows a typical STM image of C_60_ on Au(111) surface in aqueous solution prepared by the transfer of L films. The structures had the same arrangement at room temperature when adlayers were prepared by vapor deposition [57,58]. At 4.5 K on Au(111), some corrugations were observed in the molecular features (Figure 12B) [58], which indicated the internal structures could be revealed by controlling temperature and therefore inhibiting rotation. The internal structures of adsorbed C_60_ and its temperature dependency were also observed on other substrates such as Au(110) [62]. Molecules in 2D self-assembled structures can clearly rotate at room temperature, even though they may not move laterally on the surface.

**Figure 13:**
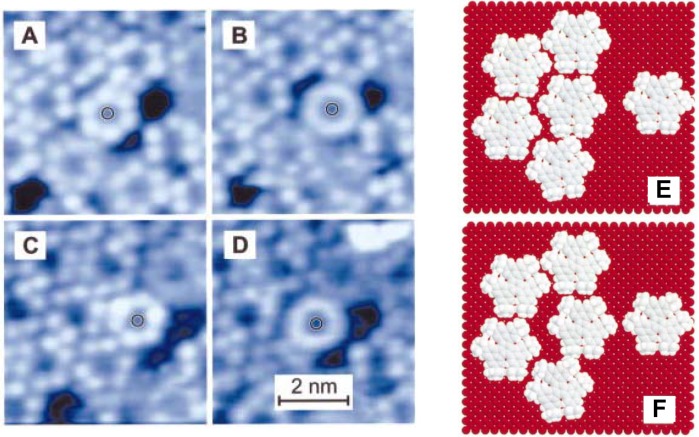
UHV-STM images (A-D) and tentative models (E and F) of propeller-shaped hexa-*tert*-butyl decacyclene on Cu(100) at room temperature. Reproduced from reference [63] by permission of the American Association for the Advancement of Science.

Molecules with lower symmetry than C_60_ cannot rotate in the ordered phase of their 2D supramolecular structures, despite single isolated molecules not being observed (under the same conditions) when the 2D structures are observed by STM at room temperature. Gimzewski and coworkers reported that the propeller-shaped molecule, hexa-*tert*-butyl decacyclene, formed hexagonal structures (2D van der Waals crystal) on Cu(100) at room temperature prepared by the vapor deposition with monolayer coverage [63]. In these structures, molecules could be stopped by the intermolecular steric interactions, allowing observation of the internal structure. At sub-monolayer coverage, the molecules were extremely active on the surface due to the weak interaction with the substrate, and the molecules located at out of the phase positions rotated. Figure 13 shows the sequential images and tentative models of the propeller molecules on Cu(100) surface. When the propeller molecule was not located in phase (*i.e.*, not at a symmetric site), the features were torus (Figure 13B and D). When the molecule was located in phase (*i.e.*, on a symmetric site), the internal structures was clearly apparent (Figure 13E and F). Such drastically altered motion was the first evidence that a room-temperature molecular rotor and supramolecular bearing could be obtained similar to a real-world mechanical system.

**Figure 14:**
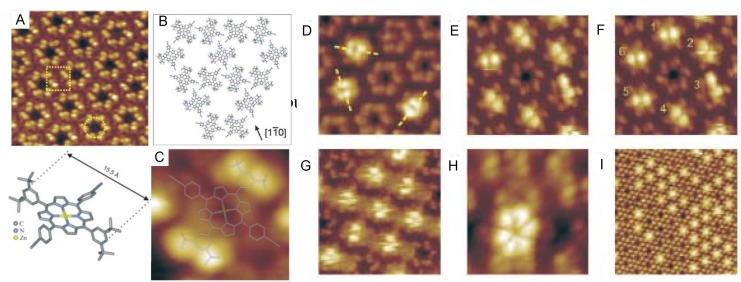
Sequential STM images (A, C-I) and the schematic illustration (B) of a porphyrin derivative on Cu(111) at different temperatures. Reproduced with permission from reference [64]. Copyright Wiley-VCH Verlag GmbH & Co. KGaA.

Molecular rotation at locations out of the ordered adlayer phase can be controlled by temperature. Guest molecules located in hollows of 2D supramolecular structures may undergo rotation at room temperature because they are not bound in the hollows or to the substrate. Jung and coworkers reported porphyrin networks and their thermal stability [64]. 2D structures of a porphyrin derivative on Cu(111) prepared by vapor deposition were observed by STM in UHV at temperature between 77 K and 298 K. The 2D structures appeared as windmill-shapes with pores as black circles (Figure 14A), with each pore surrounded by six porphyrin molecules (Figure 14B).

Porphyrin molecules (*i.e.*, a porphyrin floating in a hollow) were also found in the pores, and were apparent as brighter spots. Their features altered drastically with temperature change (Figure 14D-H). At 77 K, the guests showed four brighter lobes (Figure 14D). Increasing the temperature to 112 K resulted in the guests’ molecular features appearing as two clear lobes (Figure 14E and F). At 115 K (Figure 14G), these bright molecular features were blurred. At 150 K (Figure 14H), the molecules appeared as six leaves, due to the slow rotation of the guest in the hollow. At room temperature (Figure 14I), the pores with guests appeared to be completely filled. These observations indicate the rotation of guest molecules in hollows at high temperature, and rotation inhibition at decreased temperature. These results illustrate the rotation and motion of isolated molecules on a surface, and could be applied as thermally induced molecular motors or switches.

**Figure 15:**
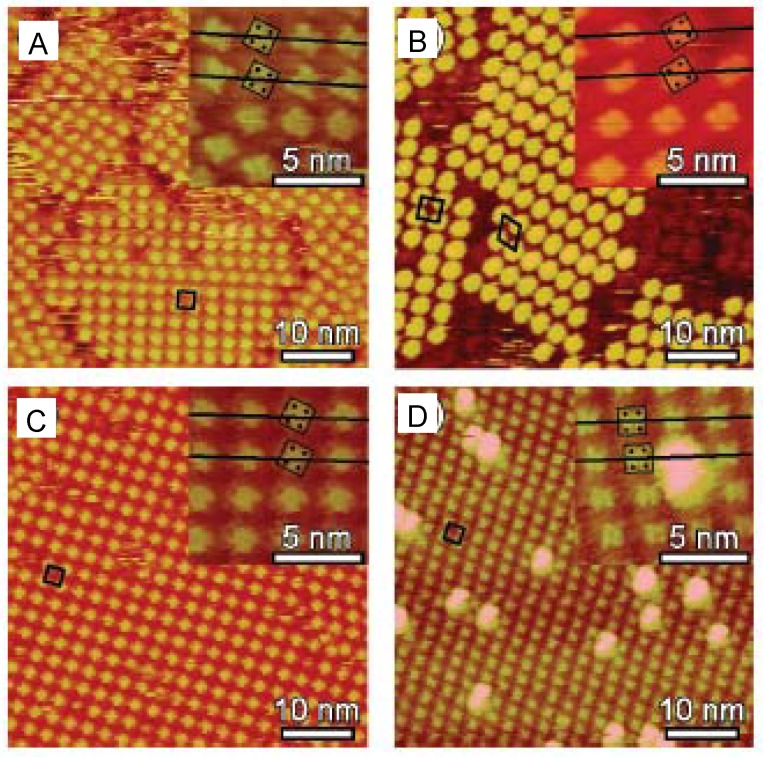
STM images of double-decker complexes at the solution–HOPG interface. Reprinted with permission from reference [74]. Copyright 2009 American Chemical Society.

As previously mentioned, molecules that float above the substrate are of interest for future molecular devices. Double-decker-type phthalocyanines and porphyrins have received much interest in supramolecular chemistry as candidates for molecular mechanics, examples of which include molecular rotors and bearings. The observation of molecular rotation of the top ligand, which is also influenced by vertical intermolecular interactions, would be interesting. The first STM investigation of double-decker phthalocyanine derivatives was reported by De Feyter and co-workers [65]. Both this group and subsequent researchers [66,67,68,69,70,71,72,73,74,75] reported that well-defined molecular features of the top ligands of double-decker phthalocyanines were not frequently observed. This was attributed to rotation of the ligand, and only a featureless spot was observed. Restricted rotation by steric hindrance or other interactions gave each molecular image the feature of legs. Miyake and co-workers recently reported the molecular motion of the top ligand for several double-decker complexes [74]. Figure 15 shows STM images of various double-decker complexes at the solution–HOPG interface.

Double-decker phthalocyanines with long-alkyl substituted top ligands had clearly apparent internal structures (Figure 15A and B), and was due to steric hindrance by the long alkyl chains. The internal structure of the double-decker phthalocyanine with an unsubstituted top phthalocyanine ligand could be observed by STM (Figure 15C). However, the internal structure of the double-decker complex consisting of a long alkyl substituted phthalocyanine and an unsubstituted porphyrin could not be observed. When the double-decker complex was prepared by mixing in solution, isolated structures with large, bright, round spots were observed (Figure 15D). The authors suggested that this might have arisen due to vertical interactions and resulting free space around the top ligand. Weak interactions between the top porphyrin ligand and the phthalocyanine may have led to molecular motion. Free space around the top ligand induced by isolation resulted in free motion, and led to the larger features observed by STM. When controlling the molecular motion of multiple component supramolecular structures, consideration must be given to the free space surrounding floating molecules, their vertical interactions and the molecules directly adsorbed molecules on the surfaces.

## 5. Conclusions

In this review, we have summarized STM observations of molecules displaying molecular dynamics in real space. The observation of molecular motion on surfaces gives us a visual understanding of the intermolecular interactions and schematic processes of self-assembly. Controlling molecular motion on surfaces will lead to the construction of supramolecular arrangements with both static and mechanical structures bearing moveable moieties. Molecular dynamics in supramolecular systems evokes a living organism. STM has many limitations such as necessity of conductivity. New innovations like high-resolution noncontact AFM and high-speed SPM will allow the observation of molecular dynamics in real space. They will lead to new discoveries in the construction of nanoscale molecular-electronic and molecular-mechanical devices or elucidation of vital phenomenon in life science.
